# Management of gastric glomus tumor: A case report

**DOI:** 10.1097/MD.0000000000016980

**Published:** 2019-09-20

**Authors:** Xingcheng Wang, Shahbaz Hanif, Binsheng Wang, Chen Chai

**Affiliations:** a Department of General Surgery, Xian Children's Hospital, Xian, Shanxi; b Department of General Surgery, First Hospital of Lanzhou University, Lanzhou, Gansu; c Department of General Surgery, The People's Hospital of Suzhou New District, Suzhou, Jiangsu, China.

**Keywords:** diagnosis, differential diagnosis, gastric glomus tumor, immunohistochemistry, treatment

## Abstract

**Rationale:**

Gastric glomus tumor (GGT) is a rare gastrointestinal tumor and its preoperative imaging features are significant to make a correct diagnosis, while the assessment of the pathological and immunohistochemical characteristics of the specimen are the main methods used for its diagnosis. This study introduces the clinical uniqueness, endoscopic ultrasonography, radiology, histology and immunohistochemistry results of a patient with GGT to discuss the imaging and clinico-pathological features, diagnosis and differential diagnosis of GGT.

**Patient concerns:**

The patient expressed a complaint concerning an “intermittent abdominal pain for 4 months”.

**Diagnoses:**

The patient was diagnosed with gastric stromal tumor according to the clinical manifestations and imaging examination before the operation. The pathological examination of an intra-operative frozen sample confirmed the benign nature of the tumor, while post-operative immunohistochemistry results indicate the presence of a GGT. The postoperative histology revealed a tumor tissue composed of irregular blood vessels and glomus cells of same size with interstitial hyaline and mucoid degeneration. Immunohistochemical staining showed positivity for SMA (+), vimentin (3+), CD 34 (vascular +), and Factor VIII (vascular +).

**Interventions:**

The tumor was completely removed by surgery.

**Outcomes:**

The patient recovered well, and was discharged from the hospital. Five months after the operation, a normal gastric mucosa was observed by gastroscopic examination.

**Lessons:**

Most of the GGTs are benign lesions, surgical resection is the preferred treatment and they result in a good prognosis. However, malignant GGT should be treated as soon as possible because of its metastatic potential and recurrence. Adjuvant radiotherapy or chemotherapy might be useful after operation.

## 1 Introduction

Glomus tumor is a mesenchymal tumor that originates from the neuromuscular arterial canal or vascular lumen. It can develop in any part of the body, mainly occurring in the surrounding soft tissues and distal limbs.^[1,2]^ Barre and Masson first described the clinical and pathological features of glomus tumor in 1924,^[3]^ and the gastric glomus tumors (GGTs) was first reported in February 1948. With the development of various diagnostic techniques, reports on GGT are rising. They accounts for approximately 1% of gastric mesenchymal tumors.^[1,4]^ Most GGTs are benign, only few cases are malignant.^[5]^ GGT and gastric submucosal tumors such as gastrointestinal stromal tumors (GISTs) and others have similar clinical manifestations and imaging features. Therefore, GGT diagnosis mainly relies on postoperative immunohistochemical findings. Endoscopy and imaging examinations have little significance in making a preoperative diagnosis.^[6]^ This article mainly reports a case of GGT and explores the endoscopic, radiological and pathological features, clinical symptoms and the diagnosis and differential diagnosis of GGT through the interpretation of the relevant literature.

## 2 Clinical data

**Figure 1:**
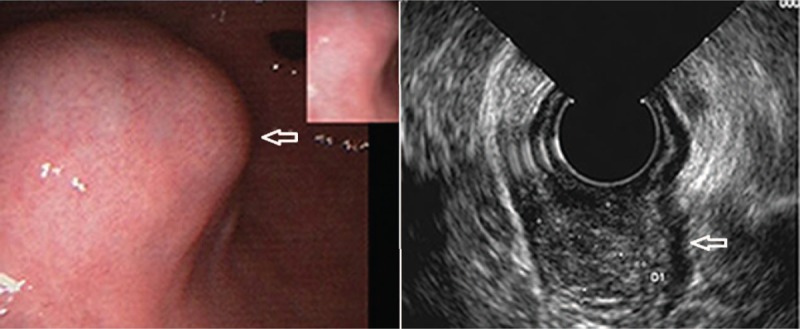
Endoscopic ultrasonography in the diagnosis of gastric stromal tumors.

**Figure 2:**
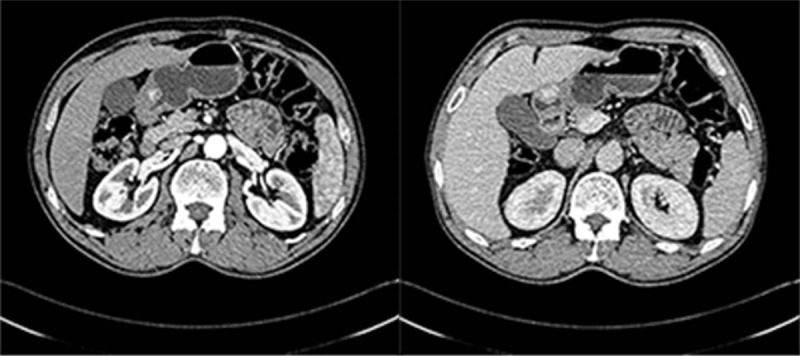
Soft tissue shadow (protruding inward) was confined in the gastric antrum with smooth edges, visible punctate calcifications and with its wide base connected to the inner layer of the gastric antrum (gastric wall). MDCT arterial phase of the tumor was significantly enhanced. The serosal surface was smooth and intact. There was no significant lymph node enlargement around the stomach.

**Figure 3:**
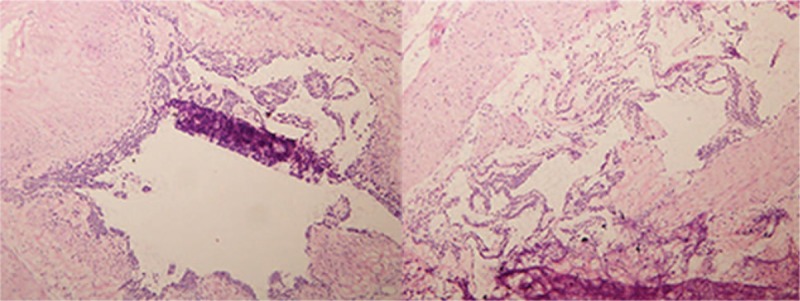
A partial resection of the distal stomach was examined, showing a mobile, spherical mass of approximately 2× 2 cm, from the medial to the serosal layer in the gastric antrum. No swollen lymph nodes were observed around the stomach. The frozen specimens showed that the tumor was confined in the mucosa with a greater curvature in the gastric antrum (hematoxylin and eosin staining; 100× magnification).

**Figure 4:**
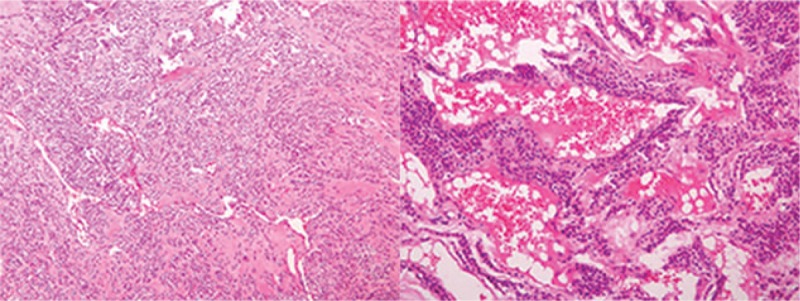
Tumor tissue consisting of irregular hyperplastic blood vessels and same sized glomus cells, with interstitial hyaline and mucoid degeneration (hematoxylin and eosin staining; A, 200× magnification; B, 100× magnification).

A 62-year-old male patient was admitted to hospital on August 31st, 2017 due to an “intermittent abdominal pain for 4 months". The patient did not show any clear cause that justified the abdominal pain, no history of nausea, vomiting, hematemesis, melena, and no clear recent change in body weight. The patient was diagnosed with gastric tumor at a local hospital while having gastroscopic examination. During hospitalization, physical examination, blood, urine, stool, liver, and kidney function tests were examined and was found to be in a normal range, with normal levels of blood coagulation factors and tumor markers. Preoperative diagnosis of gastric stromal tumor was made on the basis of the results of endoscopic ultrasonography (EUS) (Fig. 1) and the diagnosis of benign tumor of the gastric antrum was made on the basis of the results of the abdominal multi-detector dynamic computed tomography (MDCT) (Fig. 2). Laparoscopic wedge gastrectomy was performed. No malignant tissue was found during the intra-operative examination (Fig. 3). However, the microscopic examination (Fig. 4) and immunohistochemical staining of a sample derived from the surgical resected mass, led to a GGT diagnosis.

## 3 Immunohistochemistry

Immunohistochemical analysis of a sample from the surgically resected tissue led to the following results: SMA (+), vimentin (3+), CD 34 (vascular +), Factor VIII (vascular +), Ki-67 <5%, ckp (−), CD 117 (−), and CD 31 (−).

## 4 Outcome

The patient recovered well, the symptoms disappeared completely, and 5 months after operation, the gastroscopic examination showed normal gastric mucosa.

## 5 Discussion

### 5.1 Pathological result and clinical manifestations

Glomus tumor is a benign mesenchymal tumor composed of glomus cells arranged in a way that resembles the structure of an organ. WHO classification of soft tissue tumors (2002) placed it into the group of perivascular cell tumors, frequently found in the surrounding soft tissues and distal limb subcutaneous tissues.^[2]^ Paroxysmal pain is the main clinical symptom. This tumor rarely occurs in the gastrointestinal tract, nasal cavity, lung and other visceral organs. The currently known GGT was first diagnosed in 1948.^[7]^ However, it was described in 2 other patients in 1951.^[3,6]^ Since it is mainly located in the muscular layer of the gastric wall, a layer having scarce nerve supply, the clinical symptoms are atypical. The most common clinical symptoms include abdominal pain and discomfort, loss of appetite, gastrointestinal bleeding and ulcers with or without nausea and vomiting.^[8]^ Although most GGTs are benign, reports of the malignant form are rare, but cannot be ignored.^[5]^ Kirsch Baum et al first reported a malignant GGT in a 40-year-old male in 1939.^[7]^

### 5.2 Imaging examination, and histological and immunohistochemical features

MDCT, endoscopic and other imaging examinations often misdiagnose it as GIST or leiomyoma. Its endoscopic performance is often related to its growth pattern. If the GGT grows into the gastric cavity, then it is often shown as a spherical submucosal protuberant lesion with smooth edges in the gastric antrum under endoscopy. However, if it grows outside the lumen of the stomach, common endoscopy can hardly find it. In this case, EUS or abdominal MDCT is more appropriate. EUS reveals a hypoechoic region in the third or fourth layer of the gastric wall, which cannot be distinguished from other gastric submucosal tumors.^[9,10]^ In the abdominal enhanced MDCT examination, GGTs are usually located in the gastric antrum. In the arterial phase, GGT is often manifested as an inhomogeneous enhancement of the high-density shadow. In the portal venous phase, the tumor is continuously and uniformly enhanced. However, the delayed phase is a submucosal lesion characterized by the presence of the same density of the liver.^[11]^ The CT value and enhancement curves are consistent with the inferior vena cava, portal vein or descending aorta signs of vascular disease. Histologically, the tumor usually has a defined boundary, composed of prominent circular glomus cells, accompanied by aneurysmal dilatation. Immunohistochemistry shows positive vimentin and SMA, and negative S-100, CD 34, CD 117, desmin, CD 56, synaptophysin, chromogranin, neuron specific enolase and cytokeratin.^[12]^

### 5.3 Diagnosis

GGT mainly occurs in the mucosa or muscular layer of the gastric antrum, rarely occurs in the gastric fundus or body. There are several diagnostic examinations, although not perfect enough for making an accurate diagnosis: 1- Endoscopic examination, which commonly shows only a spherical submucosal protuberant lesion with a smooth edge in the gastric antrum, thus, without a specific diagnostic significance;^[7]^ 2- EUS, which shows a heterogeneous hypoechoic area that cannot be distinguished from other possible lesions such as GIST;^[7]^ 3- GGT by abdominal MDCT usually reveals it in the gastric antrum. Arterial phase scan often shows the inhomogeneous enhancement of the high-density shadow. Coronal portal phase CT image shows a sustained uniform enhancement of the tumor. Delayed phase scan shows a submucosal lesion characterized by the presence of the same density of the liver. Despite the presence of certain characteristics, it is still difficult to distinguish GGT with other features. 4- At present, some authors believe that endoscopic ultrasound-guided fine needle aspiration biopsy can diagnose the GGT before operation, but still a lot of misdiagnoses are made. The main reason is that the endoscopic ultrasound-guided needle is not one hundred percent accurate to evaluate the lesion tissue, and the tumor bleeding may affect the judgment.^[3,10]^ Under these difficulties in making a correct diagnosis, only the postoperative pathological and immunohistochemical diagnosis of GGT is still recognized as the gold standard. Although almost all glomus tumors are benign, some malignant cases have been reported, thus the diagnostic criteria have been proposed: 1- Deeper location, in the lower part of the fascia or in the solid organs, with a tumor diameter of more than 2 cm; 2- presence of atypical mitoses; 3- presence of nuclear pleomorphism, moderate to high nuclear grade, mitotic figures (5/50 HPF).^[3,13]^ However, although the average size of GGT is 2 cm, a lot of benign GGT are larger than 2 cm. There are also reports of very low mitosis (1-3/50 HPF) in GGT metastasis.^[3,13]^ Therefore, the diagnostic standards described above for malignant tumors should be further evaluated to conclude that they can be valid standards in the GGT diagnosis.

### 5.4 Differential diagnosis

GGT can be misdiagnosed with other gastric submucosal tumors originating from the mesenchyme, such as GIST, carcinoid tumors, neurilemomas and hemangiomas of the stomach and some other rare tumors. The gastric submucosal tumor belongs to non-epithelial mesenchymal tumors, mainly originating from the submucosa of the stomach or gastric muscular layer, usually covered with mucosa, and account for approximately 0.1% to 1% of the gastrointestinal tumors. The most common diagnostic modality used for gastric submucosal tumors are EUS and MDCT. EUS can be used to determine the origin of the tumor, which is generally seen as a hypoechoic tumor located in the third or fourth layer. Endoscopic ultrasound-guided biopsy is an accepted method for the diagnosis of submucosal lesions, while MDCT is used to determine tumor characteristics by contrast enhancement.^[15]^

- GIST is the most common gastric submucosal tumor, while many submucosal tumors are confused with GIST. The MDCT arterial phase scan in GIST shows a small or smooth structure on the edge of the tumor, which is characterized by a strong heterogeneity in the arterial phase, often with cystic degeneration, necrosis and hemorrhage.^[16]^ The MDCT arterial phase scan of GGT results in images different from the ones resulting from a gastric stromal tumor. The degree of enhancement is significantly higher than that of gastric stromal tumor. GIST shows a lack of dilated capillaries in the pathological examination, and immunohistochemistry shows positive CD117, CD 34 and DOG-1 in tumor cells.^[2,16]^ However, the results of GGT immunohistochemistry usually reveal positive vimentin, SMA, and negative CD 34, CD 117, DOG-1.
- Gastric carcinoids can occur in any part from the cardia to the pylorus, and invade the gastric layers (muscularis externa, serosa) or adjacent tissues. It is always accompanied by lymph node invasion and distant metastasis, but GGT is usually a single benign tumor occurring in the gastric antrum, with no metastasis or invasion of surrounding tissues. Carcinoids usually have a specific structure of nuclear staining and positive neuroendocrine markers that can be used to distinguish between gastric carcinoid and GGT.^[17]^
- Gastric hemangiomas are not true tumors; they belong to the vascular malformation, thus, CT features visible inside the tumor vein stone, can allow the differentiation from GGT.
- Gastric schwannoma mainly occurs in the body of the stomach, followed by a localization in the gastric fundus, while it is rarely localized in the gastric antrum. CT scan shows inhomogeneous low density, and cystic degeneration of the tumor. However, GGT mainly occurs in the gastric antrum, and CT scan shows no cystic degeneration.
- Paragangliomas occur in the retroperitoneum, while rarely occur in the stomach. Immunohistochemical staining shows positive synaptophysin, chromogranin A, and S-100 protein. Thus, it can be distinguished from GGT.^[18]^
- Another rare tumor, the hemangiopericytoma, should also be taken into account. The histological characteristics consist of spindle cells with elongated nuclei, and the immunohistochemical staining usually results in a negative SMA, while glomus tumor appears as circular endothelial cells with the regular elliptic or circular nucleus.^[19]^

### 5.5 Treatment and follow-up

Most of the GGTs are benign, can be cured by surgery, and with the development of various endoscopic techniques, lesions can be treated by endoscopic resections. Our patient's tumor was completely removed by surgery. No recurrence or metastasis was observed after 6 months of follow-up.

## 6 Conclusion

GGT is rare, and previous studies indicate that it is more likely occurring in the gastric antrum of adults of any age with a significant female predominance, rarely reported in other parts of the stomach.^[2,14,20,21]^ The existing auxiliary examinations, such as the imaging examination and endoscopic ultrasound-guided fine needle aspiration have an important significance in the diagnosis and treatment of GGT. However, its final and correct diagnosis depends on the postoperative pathological and immunohistochemical analysis. Laparoscopic wedge resection of the stomach is considered as the first-line treatment.^[7]^ Clinical treatment should be based on the specific location and size of the tumor. In addition, a literature report shows that GGT can be treated with EUS; however, GGT contains abundant blood vessels, with high risk of intra-operative bleeding, and many primary hospitals in our country are unaware of this endoscopic treatment technique, thus, surgical treatment is preferred.^[22]^ Due to the lack of long-term follow-up, there have been no reports of patients’ prognosis and recurrence after surgery. Most of the GGT are benign lesions, can be cured by surgical resection and have a good prognosis. Several other case reports of malignant GGT are also available,^[23–25]^ accompanied by certain characteristics of recurrence and metastasis. Thus, it is necessary for the patients carrying a potential malignancy to undergo surgical resection as soon as possible, with adjuvant therapies that might be useful. However, there is not any definitive diagnostic criterion for malignant GGT, thus, a large number of observations and studies are still needed to obtain definitive diagnostic evidence.

## Author contributions

**Conceptualization:** Xingcheng Wang.

**Data curation:** Xingcheng Wang, Shahbaz Hanif, Binsheng Wang, Chen Chai.

**Formal analysis:** Xingcheng Wang, Shahbaz Hanif, Binsheng Wang, Chen Chai.

**Project administration:** Chen Chai.

**Resources:** Xingcheng Wang.

**Software:** Xingcheng Wang.

**Supervision:** Xingcheng Wang.

**Validation:** Xingcheng Wang.

**Visualization:** Xingcheng Wang.

**Writing – original draft:** Xingcheng Wang.

**Writing – review & editing:** Xingcheng Wang.

Xingcheng Wang orcid: 0000-0002-6980-9241.
